# Hsa-mir-499 rs3746444 polymorphism and cancer risk: a meta-analysis

**DOI:** 10.7555/JBR.26.20110122

**Published:** 2012-04-16

**Authors:** Peng Zou, Lin Zhao, Haitao Xu, Ping Chen, Aihua Gu, Ning Liu, Peng Zhao, Ailin Lu

**Affiliations:** a Department of Neurosurgery, the First Affiliated Hospital, Nanjing Medical University, Nanjing, Jiangsu 210029, China;; b State Key Laboratory of Reproductive Medicine, Institute of Toxicology, Nanjing Medical University, Nanjing, Jiangsu 210029, China.

**Keywords:** cancer, meta-analysis, hsa-mir-499 rs3746444, polymorphism, susceptibility, miRNAs, pre-miRNA

## Abstract

MicroRNAs (miRNAs) are gene regulators involved in numerous diseases including cancer, heart disease, neurological disorders, vascular abnormalities and autoimmune conditions. Although hsa-mir-499 rs3746444 polymorphism was shown to contribute to the susceptibility of multiple genes to cancer, the data have yielded conflicting results. Therefore, this meta-analysis was performed to provide a comprehensive assessment of potential association between hsa-mir-499 rs3746444 polymorphism and cancer risk. In this meta-analysis, a total of 9 articles regarding 10 eligible case-control studies in English (including 6134 cases and 7141 controls) were analyzed. No significant association between hsa-mir-499 rs3746444 polymorphism and overall cancer risk was demonstrated. However, an increased risk was observed in the subgroup of breast cancer patients (G allele *vs* A allele: OR = 1.10, 95% CI = 1.00-1.20; *P*_heterogeneity_ = 0.114; *I*^2^ = 53.9%) and population-based studies (G allele *vs* A allele: OR = 1.12, 95% CI = 1.00-1.25; *P*_heterogeneity_ = 0.062; *I*^2^ = 64.0%). The findings suggested an association between hsa-mir-499 rs3746444 polymorphism and increased risk to breast cancer.

## INTRODUCTION

MicroRNAs (miRNAs) are short (around 22-nt) non-coding single-stranded RNA molecules involved in both physiological and pathological processes, exerting their regulatory effects by suppressing translation or by inducing the cleavage of target RNA transcripts[1]. MiRNAs are regulators of gene expression that provide a regulation for a broad range of biological processes including cancer development, cellular differentiation, proliferation, apoptosis and metabolism[2]–[6]. MiR-499-5p, a gene with frequently increased expression in colorectal cancer, may function to promote migration and invasion of malignant cells[7]. One study has demonstrated that both the α- and β-isoform of the calcineurin catalytic subunit are direct targets of miR-499, which inhibits apoptosis of cardiomyocytes through a suppression of calcineurin-mediated dephosphorylation of dynamin-related protein-1 (Drp1)[8].

Single nucleotide polymorphisms (SNPs), the most common type of sequence variations in the human genome, contribute to human phenotypic differences[9], and sequence variations could potentially affect the processing and/or target selection of miRNAs in miRNA genes, pri-miRNAs, pre-miRNAs and mature miRNAs[10]. Landi *et al*.[11] reported seven SNPs located in seven pre-miRNA hairpin regions, which include the polymorphism within hsa-mir499. Although a sizeable number of studies have been performed to investigate the role of hsa-mir-499 rs3746444 polymorphism in several cancer types such as breast cancer[12],[13], lung cancer[14], gallbladder cancer[15], squamous cell carcinoma of the head and neck (SCCHN)[16], prostatic cancer[17], gastric cancer[18], cervical squamous cell carcinoma (CSCC)[19] and bladder cancer[20], these existing eligible studies have yielded contradictory results, needing to be investigated further. Here, we performed a meta-analysis including subgroup analysis from all eligible studies to obtain a more precise assessment of the association between hsa-mir-499 rs3746444 polymorphism and cancer risk.

## MATERIALS AND METHODS

### Publication search

**Fig. 1:**
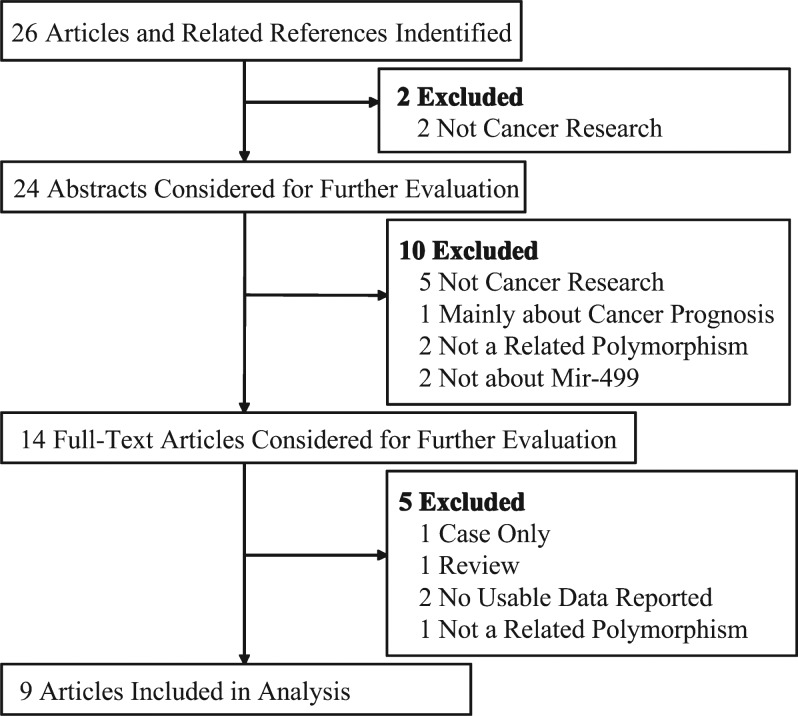
Study flow chart

We conducted a computerized literature search of PubMed, EmBase databases and Chinese National Knowledge Infrastructure (CNKI) databases (the last search was updated on August 10, 2011). The following search terms were used in isolation and combination with one another: “mir-499 or rs3746444” and “polymorphism or variant or mutation” and “cancer or carcinoma”. The search was limited to papers published in the English language. The related reference articles were reviewed to identify other potentially eligible publications. All studies matching the eligible criteria were included in our meta-analysis. ***Fig. 1*** shows the study capture procedure.

### Inclusion criteria

Studies included in the meta-analysis met the following criteria: 1) articles on hsa-mir-499 rs3746444 polymorphism and cancer risk; 2) studies using a case-control design; 3) studies containing sufficient published data for the estimation of odds ratios (ORs) with their 95% confidence interval (CI).

### Data extraction

Two investigators extracted necessary data from all eligible publications independently according to the inclusion criteria listed above. From each of the included articles the following information was abstracted: the name of first author, year of publication, country origin, ethnicity (Caucasian, Asian or others), cancer type, source of controls (population- or hospital-based controls), genotyping methods, total number of cases and controls, the number of cases and controls with hsa-mir-499 rs3746444 polymorphism genotypes, G allele frequency in controls and *P* value for Hardy-Weinberg equilibrium (HWE), respectively.

**Table 1:** Main characteristics of all studies included in the meta-analysis

First author	Year	Country	Ethnicity	Cancer type	Sourceof controls	Genotyping method	Cases	Controls	Case			Control			G alleleincontrols(%)	HWE
									AA	AG	GG	AA	AG	GG		
Hu	2008	China	Asian	Breast	PB	PCR-RFLP	1009	1093	707	258	44	816	248	29	0.14	0.06
Tian	2009	China	Asian	Lung	PB	PCR-RFLP	1058	1035	781	253	24	755	254	26	0.15	0.40
Catucci	2010	Italy	Caucasian	Breast	HB	Sequencing	756	1242	414	295	47	704	452	86	0.25	0.25
Catucci	2010	Germany	Caucasian	Breast	HB	Sequencing	823	925	536	250	37	601	290	34	0.19	0.89
Srivastava	2010	India	Caucasian	Gallbladder	PB	PCR-RFLP	230	230	112	97	21	121	94	15	0.27	0.57
Liu	2010	America	Caucasian	SCCHN	HB	PCR-RFLP	1109	1130	745	309	55	710	366	54	0.21	0.44
George	2010	India	Caucasian	Prostate	HB	PCR-RFLP	159	230	48	98	13	104	92	34	0.35	0.07
Okubo	2010	Japan	Asian	Gastric	HB	PCR-RFLP	552	697	364	151	37	466	198	33	0.19	0.05
Zhou	2011	China	Asian	CSCC	HB	PCR-RFLP	226	309	134	84	8	223	71	15	0.16	0.00
Mittal	2011	India	Caucasian	Bladder	HB	PCR-RFLP	212	250	95	92	25	121	94	35	0.33	0.02

HB: hospital-based of control; PB: population-based of control; PCR-RFLP: polymerase chain reaction-restriction fragment length polymorphism; HWE: Hardy -Weinberg equilibrium; SCCHN: squamous cell carcinoma of the head and neck; CSCC: cervical squa-mous cell carcinoma.

### Statistical analysis

We first assessed HWE for the controls in each study. The strength of the association between polymorphism and cancer risk was assessed by ORs with 95% CIs. The statistical significance of the summary OR was determined by *Z* test (*P* < 0.05 was considered statistically significant). The pooled ORs were calculated and used for comparisons between two homozygotes (GG *vs* AA), two heterozygotes (AG *vs*. AA), dominant models (GG+AG *vs* AA), recessive models (GG *vs* AG+AA) and the allele contrast models (G *vs* A), respectively. Subgroup analyses were also performed by cancer types (a cancer type with less than three individual studies was combined into other cancer groups), ethnicity and source of controls. Inter-study heterogeneity was estimated using a chi-square-based *Q*-test. The pooled ORs were analyzed using a random effects model (the DerSimonian and Laird method)[21] in case of a significant result (*P* < 0.05) provided by the *Q*-test, and using a fixed-effects model (the Mantel-Haenszel method)[22] if insignificance (*P* > 0.05) was found. The *I*^2^ (*I*^2^ = 100% × (*Q-df*)/*Q*) statistic was then used to quantitatively estimate heterogeneity, where *I*^2^ < 25%, 25-75% and >75% represent low, moderate and high inconsistency, respectively[23],[24]. Additionally, sensitivity analyses were performed by omitting each study to reflect the influence of the individual data on the summary ORs. Finally, publication bias of literatures was estimated using the Begg's funnel plot and Egger's test (*P* < 0.05 was considered a significant publication bias)[25]. All statistical analyses were performed with the software Stata (Version 11; Stata Corporation, College Station, Texas, USA), and all tests were two-sided.

## RESULTS

### Eligible studies

Through an extensive search, 9 articles regarding 10 case-control studies in English (including 6134 cases and 7141 controls) met the inclusion criteria. In the study by Catucci *et al*.[13] the genotype data were presented separately in a German study and an Italian study, which were thus considered as two separate studies for this meta-analysis. Of the 10 studies, 3 focused exclusively on breast cancer[12],[13] and 7 focused on other cancers[14]–[20]. The 10 studies collected in this meta-analysis included 4 studies on Asians[12],[14],[18],[19] and 6 studies on Caucasians[13],[15]-[17],[20]. The population-based[12],[14],[15] and hospital-based design[13],[16]-[20] were used in 3 and 7 studies, respectively. Several genotyping methods including polymerase chain reaction-restriction fragment length polymorphism (PCR-RFLP)[12],[14]-[20] and DNA sequencing[13] were also used. The genotype distributions in the control groups were generally consistent with the criteria of HWE except one study[19]. ***Table 1*** presents the main characteristics of eligible studies in the meta-analysis.

### Evidence synthesis

**Fig. 2:**
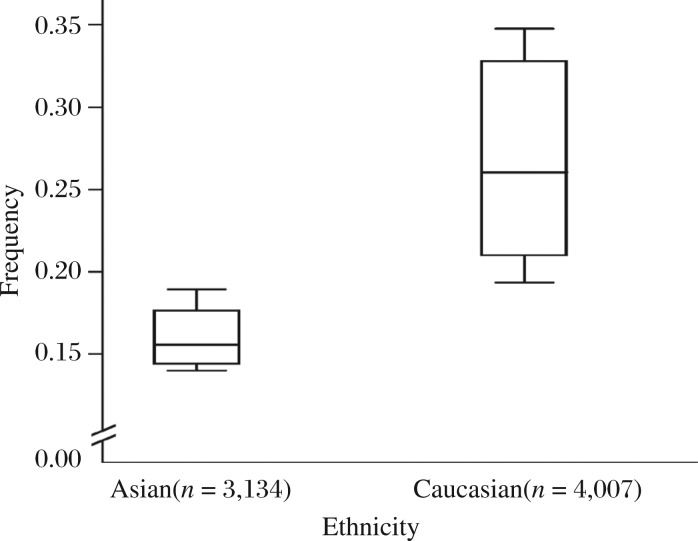
Frequencies of the variant alleles among controls stratified by ethnicity

We observed a wide variation of G allele frequencies across different ethnicities. For Caucasians, the G allele frequency was 26.7% (95% CI = 20.2%-33.2%), significantly (*P* = 0.01) higher than that in Asian populations (16.0%, 95% CI = 12.5%-19.5%) (***Fig. 2***).

**Table 2:** Stratified analyses of the hsa-mir-499 rs3746444 polymorphism on cancer risk

Variables	*n*^a^	Cases/controls	G *vs* A			GG *vs* AA			AG *vs* AA		
			OR(95%CI)	*P*^b^	*I^2^*(%)	OR(95%CI)	*P*^b^	*I^2^*(%)	OR(95%CI)	*P*^b^	*I^2^*(%)
Total	10	6,134/7,141	1.07(0.98-1.17)^c^	0.037	49.7	1.11(0.94-1.30)	0.473	0.0	1.14(0.97-1.34)^c^	0.000	74.3
Cancer type											
Breast cancer	3	2,588/3,260	1.10(1.00-1.20)	0.114	53.9	1.19(0.93-1.53)	0.124	52.0	1.09(0.97-1.22)	0.327	10.6
Other cancer	7	3,546/3,881	1.02(0.94-1.11)	0.057	51.0	1.05(0.85-1.29)	0.698	0.0	1.20(0.93-1.55)^c^	0.000	81.5
Ethnicity											
Asian	4	2,845/3,134	1.15(0.98-1.36)^c^	0.042	63.4	1.30(0.99-1.72)	0.254	26.2	1.18(0.92-1.51)^c^	0.007	75.3
Caucasian	6	3,289/4,007	1.00(0.93-1.08)	0.310	16.1	1.01(0.83-1.24)	0.780	0.0	1.13(0.90-1.41)^c^	0.001	76.9
Source of controls											
PB	3	2,297/2,358	1.12(1.00-1.25)	0.062	64.0	1.36(0.99-1.88)	0.192	39.4	1.08(0.95-1.23)	0.309	14.7
HB	7	3,837/4,783	1.03(0.95-1.10)	0.096	44.3	1.03(0.85-1.24)	0.776	0.0	1.19(0.94-1.50)^c^	0.000	81.6

HB: hospital-based of control; PB: population-based of control. ^a^Number of comparisons. ^b^*P* value of *Q*-test for heterogeneity test. ^c^Random-effects model was used when *P* value for heterogeneity test <0.05; otherwise, fix-effects model was used.

**Fig. 3:**
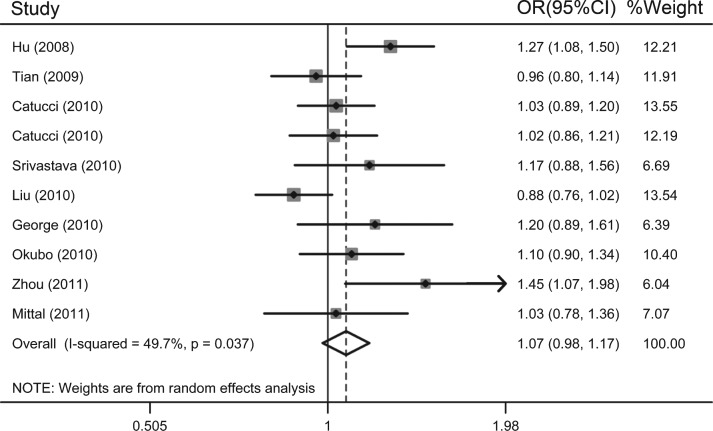
Meta-analysis of the association between hsa-mir-499 rs3746444 polymorphism and susceptibility to cancer under allele contrast (G *vs* A).

As shown in ***Table 2***, no significant association between hsa-mir-499 rs3746444 polymorphism and cancer risk was demonstrated in overall analysis (GG *vs* AA: OR = 1.11, 95% CI = 0.94-1.30; *P*_heterogeneity_ = 0.473; *I*^2^ = 0.0%; AG *vs* AA: OR = 1.14, 95% CI = 0.97-1.34; *P*_heterogeneity_ = 0.000; *I*^2^ = 74.3%; G *vs* A: OR = 1.07, 95% CI = 0.98-1.17; *P*_heterogeneity_ = 0.037; *I*^2^ = 49.7%). Similarly, insignificant effects were also found under the dominant and recessive model. ***Fig. 3*** shows the forest plot of the association between cancer risk and hsa-mir-499 rs3746444 polymorphism under allele contrast (G allele *vs* A allele).

### Subgroup analyses

Subgroup analyses were performed of hsa-mir-499 rs3746444 polymorphism by cancer type, showing that the rs3746444 polymorphism was associated with elevated risk in breast cancer (G *vs* A, OR = 1.10, 95% CI = 1.00-1.20; *P*_heterogeneity_ = 0.114; *I*^2^ = 53.9%) rather than in other cancer types (***Table 2***). When stratified by ethnicity, the results showed no significant association between hsa-mir-499 rs3746444 polymorphism and cancer risk among Asians and Caucasians in all comparison models tested (***Table 2***). In the subgroup analysis by the source of controls, significantly increased risk was found in the population-based studies (G *vs* A, OR = 1.12, 95% CI = 1.00-1.25; *P*_heterogeneity_ = 0.062; *I*^2^ = 64.0%), not in the hospital-based studies (***Table 2***).

### Test of heterogeneity

There was significant heterogeneity in three genetic models (AG *vs* AA: *P*_heterogeneity_ = 0.000; *I*^2^ = 74.3%; dominant model-AG+GG *vs* AA: *P*_heterogeneity_ = 0.001; *I*^2^ = 68.0%; G *vs* A: *P*_heterogeneity_ = 0.037; *I*^2^ = 49.7%). In the subgroup analyses of cancer types, ethnicity and source of controls, heterogeneity was not observed any longer in breast cancer (AG *vs* AA: *P*_heterogeneity_ = 0.327; *I*^2^ = 10.6%; dominant model-AG+GG *vs* AA: *P*_heterogeneity_ = 0.229; *I*^2^ = 32.1%; G *vs* A: *P*_heterogeneity_ = 0.114; *I*^2^ = 53.9%) and other cancer (G *vs* A: *P*_heterogeneity_ = 0.057; *I*^2^ = 51.0%), in Caucasian populations (G *vs* A: *P*_heterogeneity_ = 0.310; *I*^2^ = 16.1%), and in population-based (AG *vs* AA: *P*_heterogeneity_ = 0.309; *I*^2^ = 14.7%; dominant model-AG+GG *vs* AA: *P*_heterogeneity_ = 0.136; *I*^2^ = 49.9%; G *vs* A: *P*_heterogeneity_ = 0.062; *I*^2^ = 64.0%) and hospital-based studies (G *vs* A: *P*_heterogeneity_ = 0.096; *I*^2^ = 44.3%).

**Fig. 4:**
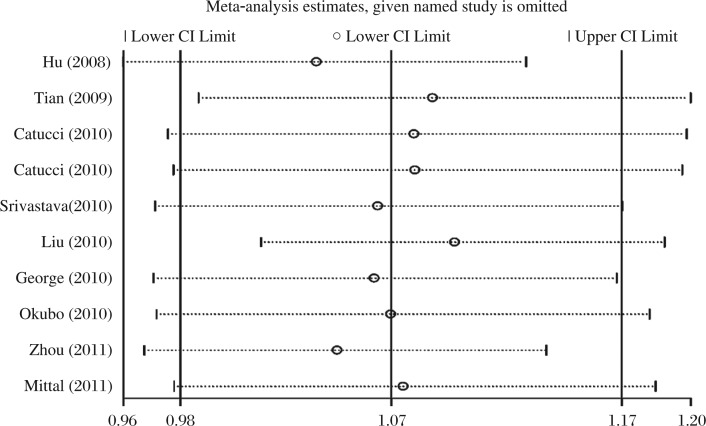
The influence of individual studies on the summary odds ratio (OR). The middle vertical axis indicates the overall OR and the two vertical axes indicate its 95% confidence interval (CI). Every hollow round indicates the pooled OR when the left study was omitted in this meta-analysis. The two ends of every broken line represent the 95% CI.

### Sensitivity analysis

In most of the studies, the frequency distributions of genotypes in the controls are generally consistent with the principles of HWE except one research conducted by Zhou *et al*.[19]. Removal of this study did not significantly change the corresponding pooled ORs. None of the pooled ORs were significantly affected by any single study, suggesting robustness of our results (***Fig. 4***).

### Assessment of bias

**Fig. 5:**
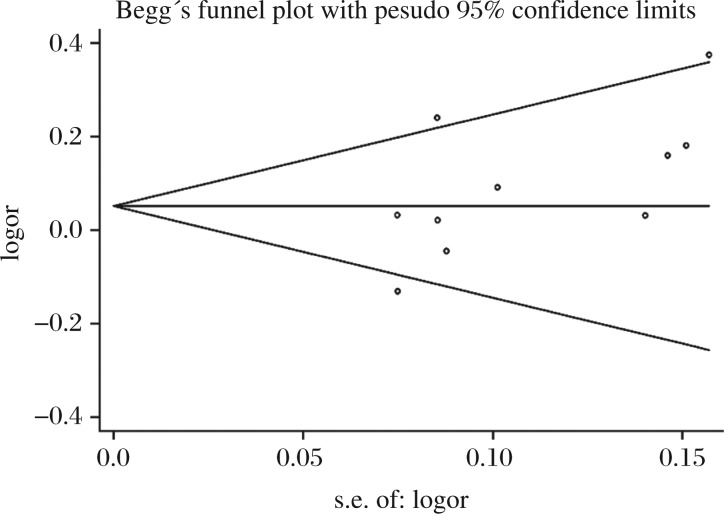
Begg's funnel plot for publication bias test (G *vs* A).

We used Begg's funnel plot and Egger's test to assess the publication bias. The graphical funnel plots for the comparison of the G allele and the A allele appeared to be symmetrical (***Fig. 5***). The results of Egger's test did not show any evidence of publication bias (*t* = 1.90, *P* = 0.095) for the G *vs* A allele contrast model.

## DISCUSSION

MiRNAs are short nucleotide RNAs that may influence mRNA stability and translation[26]. Recent studies have demonstrated that miRNAs can act as either tumor suppressors or oncogenes for cancers[26]–[29] and are associated with many other diseases including heart disease, neurological disorders, and autoimmune conditions. SNPs are the most common source of genetic polymorphism in the human genome. Various genetic association studies have explored the association between pre-miRNA polymorphisms and cancer risk. The SNP rs3746444 located in its corresponding 3p mature miRNAs regions may influence both the binding of 3p mature miRNAs to target mRNAs and pre-miRNA maturation of 5p and 3p miRNAs[12]. Srivastava *et al*.[15] reported that genetic variations in the miR-499 gene could alter PDCD4 expression levels, subsequently progressing to gallbladder epithelium transformation. In this study, to clarify controversial results from previous studies, we carried out a meta-analysis to investigate the association of hsa-mir-499 rs3746444 polymorphism with cancer. However, we failed to find any significant association between this polymorphism and cancer risk, which may be due to the limited number of studies included in this meta-analysis, the different matching criteria of the control group across studies, selection bias, and ethnic variation.

As tumor origin can influence the results from meta-analysis, we performed subgroup analyses by cancer type for hsa-mir-499 rs3746444 polymorphism. Our meta-analysis on the available studies showed that this polymorphism was associated with the risk of breast cancer, suggesting that this polymorphism might be biologically functional in the pathogenesis of this malignancy. Some factors might account for these. First, hsa-mir-499 rs3746444 polymorphism may exert varying effect on the mechanisms of carcinogenesis, which may differ in different cancers. Second, hsa-mir-499 rs3746444 polymorphism detected in an invasive breast cancer cell line[30] suggested its relevance for promoting breast cancer progression. In the stratified analysis by ethnicity, no significantly increased cancer risks were found in Asians or Caucasians. The null result may be due to the limited number of studies, which had insufficient statistical power to detect a significant effect. Further studies are required to examine this association.

The results of meta-analysis often depend on control selection procedures. According to the source of controls, significantly increased risks were observed in the population-based studies. This may be due to the fact that the population-based controls might be typically representative of the general population and be better to reduce biases in such genetic association studies. Since there are only 3 population-based studies available, the data presented in this meta-analysis have limited power to reveal a reliable association. Further meta-analysis in the population-based studies subgroup with a larger sample size is necessary to be performed to examine this association.

Our meta-analysis should be interpreted within the context of its limitations. First, the total number of studies was too small to perform subgroup analyses. Second, our results were obtained based on the unadjusted estimates due to a lack of the genotype information stratified for the main confounding variables in the original papers. More accurate OR should be corrected by age, gender, smoking status, alcohol consumption and other exposure factors. Third, in this meta-analysis, the effect of gene-gene and gene-environment interactions was not addressed.

In conclusion, this meta-analysis provided evidence that hsa-mir-499 rs3746444 polymorphism is not associated with cancer risk. However, stratified analysis indicated a significantly increased risk of breast cancer (G *vs* A: OR = 1.10, 95% CI = 1.00-1.20; *P*_heterogeneity_ = 0.114; *I*^2^ = 53.9%) and cancer in population-based studies (G *vs* A: OR = 1.12, 95% CI = 1.00-1.25; *P*_heterogeneity_ = 0.062; *I*^2^ = 64.0%). Further larger, preferably prospective studies are required to evaluate the role of hsa-mir-499 rs3746444 polymorphism in cancer risk.
